# Trends in Kampo Medicine Usage as Supportive Care During Anticancer Drug Treatment in Japanese Patients: A Nationwide Cohort Analysis from Fiscal Years 2015 to 2021

**DOI:** 10.3390/curroncol32020100

**Published:** 2025-02-10

**Authors:** Hiroaki Ohta, Takeo Yasu

**Affiliations:** Department of Medicinal Therapy Research, Education and Research Unit for Comprehensive Clinical Pharmacy, Meiji Pharmaceutical University, Tokyo 204-8588, Japan; d246951@std.my-pharm.ac.jp

**Keywords:** anticancer drug, Kampo medicine, herbal medicine, complementary and alternative medicine, trend

## Abstract

The adverse effects of anticancer drugs significantly impact the quality of life of patients undergoing chemotherapy, necessitating evidence-based supportive therapies. In Japan, Kampo medicines, traditional Japanese herbal therapies used for relief of various symptoms, have been widely used as complementary and alternative treatments for cancer, despite limited evidence regarding their efficacy and safety. Thus, we investigated the actual use of Kampo medicines as supportive care in patients undergoing anticancer drug treatment and evaluated the trends in prescription according to year. We analyzed 89,141 cancer drug therapy cases registered in the Japan Medical Data Center database between April 2014 and July 2022, excluding those with a history of Kampo medicine prescriptions before the first prescription of antineoplastic drugs. We assessed the trends in prescription according to sex, age group (<50, 50–74, and ≥75 years), and cancer type subgroup using the Cochran–Armitage trend test. Approximately 23.7% of patients were prescribed Kampo medicines during anticancer drug treatment. Since 2014, a decrease in the prescription of Kampo medicines during anticancer treatment has been observed regardless of sex, age, or cancer type. These findings suggest that recent negative reports on the efficacy and safety of Kampo medicines in cancer care may have influenced this trend.

## 1. Introduction

Malignant neoplasms are the leading cause of death worldwide [1]. Despite advances in cancer treatments, such as molecularly targeted drugs and immune checkpoint inhibitors, these therapies often result in significant side effects. Consequently, an increasing demand exists for supportive care to improve the quality of life (QOL) of patients with cancer [2]. Supportive care is essential for managing symptoms and maintaining QOL throughout the cancer journey.

Guidelines for supportive care emphasize that supportive care aims to improve and maintain the quality of life (QOL) of cancer patients while addressing the physical, emotional, and psychological challenges they face [3]. The effectiveness of supportive care interventions during chemotherapy has been demonstrated in several studies [4,5,6], highlighting their growing recognition and adoption as a standard component of comprehensive cancer treatment.

Recently, the use of complementary and alternative medicine (CAM) in supportive care has increased considerably [7]. According to the National Center for Complementary and Integrative Health, CAM modalities are generally classified into five categories depending on their primary therapeutic input: nutritional (e.g., probiotics, dietary supplements), physical (e.g., heat/cold therapies, massage), psychological (e.g., spiritual practice, mindfulness), combinations such as psychological and physical (e.g., tai chi, yoga) or psychological and nutritional (e.g., mindful eating and herbs), and other complementary health approaches [8]. The role of CAM in cancer supportive care is recognized for its application in alleviating the side effects of invasive treatments, such as chemotherapy and radiation therapy. Moreover, it is employed for its perceived benefits in promoting health, managing disease symptoms, preventing illness, and improving immune function [9,10].

Kampo medicine, a traditional Japanese herbal medicine, is one of the most frequently used forms of CAM in Japan [11,12,13,14,15]. It consists of natural substances combined into formulas and has been covered by Japan’s National Health Insurance System since 1967. Currently, 148 types of Kampo medicines are listed in the pharmaceutical price standard [16]. For example, Daikenchuto, one type of Kampo medicine, is used for constipation and intestinal obstruction [17,18], Goshajinkigan is used for lower back pain [19], and Shakuyakukanzoto is used for skeletal muscle cramps and intestinal cramps [20]. Additionally, Kampo medicines are commonly used as supportive care for managing side effects of cancer treatments, such as anorexia, peripheral neuropathy, and cachexia [21,22]. However, their utility is limited by the lack of high-quality evidence, as clinical trials typically conducted for pharmaceuticals (e.g., Phase I, II, and III trials) have not yet been performed [23].

Previous studies have reported the widespread use of Kampo medicine in Japan; however, its efficacy and safety have not been consistently validated. While some patients have reported experiencing benefits in managing symptoms related to chemotherapy [21,22], robust evidence remains lacking. Despite these limitations, Kampo medicine continues to play a significant role in clinical practice in Japan.

Considering the limited evidence supporting Kampo medicine use, a pressing need exists to evaluate its role in cancer supportive care. This study aims to investigate the prescription patterns of Kampo medicines used among patients with cancer undergoing chemotherapy on a national scale. By analyzing these patterns, we hope to contribute to the foundation of evidence supporting Kampo medicine’s role in cancer supportive care in Japan.

## 2. Materials and Methods

### 2.1. Study Design and Databases

This retrospective study utilized data from the Japan Medical Data Center (JMDC) hospital-based administrative claims database, which is a private database provided by JMDC Inc. (Tokyo, Japan, https://www.jmdc.co.jp) [24]. The database consisted of claims (for hospitalization and outpatient treatment), assessment forms for the combination of diagnosis procedures, and clinical laboratory test values. As of September 2023, medical data were collected from 860 medical institutions. The disease class was based on the 10th revision of the International Statistical Classification of Diseases and Related Health Problems, as defined by the World Health Organization. We extracted data on age, sex, and cancer type based on the 10th revision of the International Statistical Classification of Diseases and Related Health Problems codes and concomitant medications from the JMDC database. Although the JMDC database is a private database, comparisons with the National Database (NDB) managed by the Japanese government have shown similar distributions of age groups and patient distributions based on ICD-10 codes [24]. This indicates that research results based on the JMDC database can be considered representative of the Japanese population. This study was conducted in accordance with the principles of the Declaration of Helsinki and approved by the Ethics Committee of Meiji Pharmaceutical University (No. 202455).

### 2.2. Data Collection

We obtained data from the JMDC medical institution database for patients who were prescribed anticancer drugs (ATC codes L01 and L02) between April 2014 and July 2022 [25]. In this study, the duration of anticancer drug treatment was defined as starting from the initial prescription date of the anticancer drug and ending six months (183 days) after the final prescription date. The inclusion criteria were patients with data available for at least one year before the initial prescription of anticancer drugs and no history of Kampo medicine prescriptions before the initial anticancer drug prescription. Cancer types were categorized according to the global cancer statistics provided by GLOBOCAN, including cancers of the lip, oral cavity (C00–C06), salivary glands (C07–C08), oropharynx (C09–C10), nasopharynx (C11), hypopharynx (C12–C13), esophagus (C15), stomach (C16), colon (C18–C21), liver (C22, including intrahepatic bile ducts), gallbladder (C23), pancreas (C25), larynx (C32), and lung (C33–C34, including trachea and bronchus); melanoma of the skin (C43); non-melanoma skin cancer (C44, excluding basal cell carcinoma for incidence); mesothelioma (C45); Kaposi sarcoma (C46); cancers of the female breast (C50), vulva (C51), vagina (C52), cervix uteri (C53), corpus uteri (C54), ovary (C56), penis (C60), prostate (C61), testis (C62), kidney (including renal pelvis, C64–C65), bladder (C67), brain, central nervous system (C70–C72), and thyroid (C73); Hodgkin lymphoma (C81); non-Hodgkin lymphoma (C82–C86, C96); multiple myeloma (C88 and C90, including immunoproliferative diseases); and leukemia (C91–C95) [1]. Patients whose cancer type could not be identified were also excluded. The baseline patient characteristics included age, sex, and cancer type. Patients who were first prescribed one of the 148 Kampo medicine extract formulations available in Japan after the prescription of anticancer drugs were categorized into the Kampo prescription group. In contrast, those without a history of Kampo medicine prescriptions were classified into the non-Kampo prescription group.

The prescription status of Kampo medicines was evaluated based on the types of Kampo medicines used after the prescription of anticancer drugs and the trends in annual prescription proportions. The fiscal year in Japan is 12 months, from 1 April in one calendar year to 31 March in the following year. In this study, all the study years are presented as fiscal years. The annual prescription proportion was defined as the percentage of patients who were prescribed Kampo medicines at least once during the anticancer treatment period among those initially prescribed anticancer drugs within a given year.

### 2.3. Statistical Analysis

The baseline characteristics of the patients in the Kampo prescription and non-prescription groups are presented as n (%) for categorical variables and median (interquartile range) for continuous variables. We compared the characteristics of patients with and without Kampo medicine prescriptions using the absolute standardized difference, where a value >0.10 indicates a significant difference. Trend analyses for categorical variables were conducted using the Cochran–Armitage test. Annual trends in Kampo medicine prescriptions were further analyzed within the following clinically relevant subgroups: age (<50, 50–74, or ≥75 years), sex (male or female), cancer type, and the ten most frequently prescribed Kampo medicines during the anticancer treatment period. All statistical analyses were two-tailed, and statistical significance was defined as *p* < 0.05. All statistical analyses were performed using EZR (version 2.9-1; Saitama Medical Center, Jichi Medical University, Saitama, Japan) [26].

## 3. Results

### 3.1. Patient Characteristics

**Figure 1:**
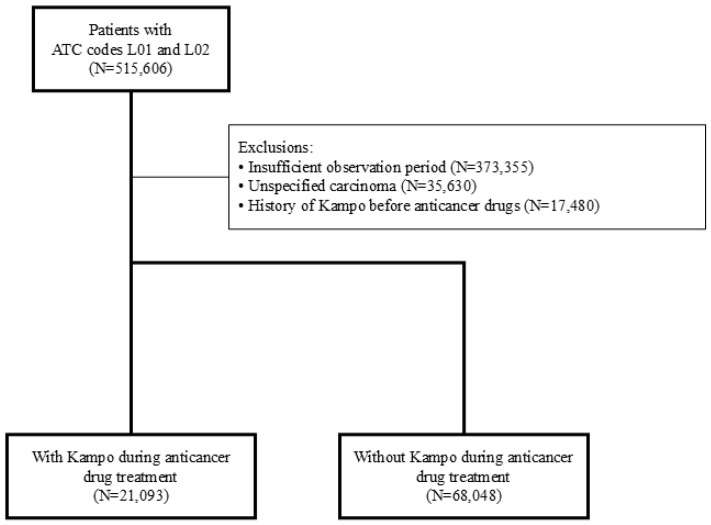
Flowchart of study patients.

A total of 515,606 patients prescribed anticancer drugs were registered in the JMDC database between April 2014 and July 2022. Of these, 89,141 met the study inclusion criteria after excluding 426,465 patients (Figure 1). Baseline patient characteristics are shown in Table 1. The cohort was 58.9% male (52,532) with a median age of 72 years (interquartile range 64–79 years). The most common cancer types were prostate (13,070 patients, 14.7%), breast (13,030 patients, 14.6%), colon (10,693 patients, 12.0%), lung (10,655 patients, 12.0%), bladder (9484 patients, 10.6%), stomach (6109 patients, 6.9%), pancreatic (4248 patients, 4.8%), liver (3323 patients, 3.7%), non-Hodgkin lymphoma (5378 patients, 6.0%), and leukemia (2064 patients, 2.3%).

### 3.2. Patients Prescribed Kampo Medicines

During the anticancer treatment period, 21,093 patients (approximately 23.7%) were prescribed Kampo medicines. Patients prescribed Kampo medicines were younger and more frequently diagnosed with carcinomas of the colon, stomach, pancreas, corpus uteri, ovary, or cervix uteri compared to those not prescribed Kampo medicines (Table 1). The top 10 Kampo medicines prescribed were Daikenchuto (4692 patients, 22.2%), Goshajinkigan (3866 patients, 18.3%), Shakuyakukanzoto (3458 patients, 16.4%), Rikkunshito (2360 patients, 11.2%), Hangeshashinto (1925 patients, 9.1%), Hochuekkito (1429 patients, 6.8%), Jyuzendaihoto (1157 patients, 5.5%), Goreisan (1153 patients, 5.5%), Yokukansan (1086 patients, 5.1%), and Kakkonto (965 patients, 4.6%). Among patients in the Kampo prescription group, the most common cancer type was colon cancer (3717 patients, 17.6%), followed by female breast (2370 patients, 11.2%), lung (2211 patients, 10.5%), stomach (1913 patients, 9.1%), and prostate (1567 patients, 6.8%) cancers (Table 2).

### 3.3. Trends in Kampo Medicine Prescriptions

**Figure 2:**
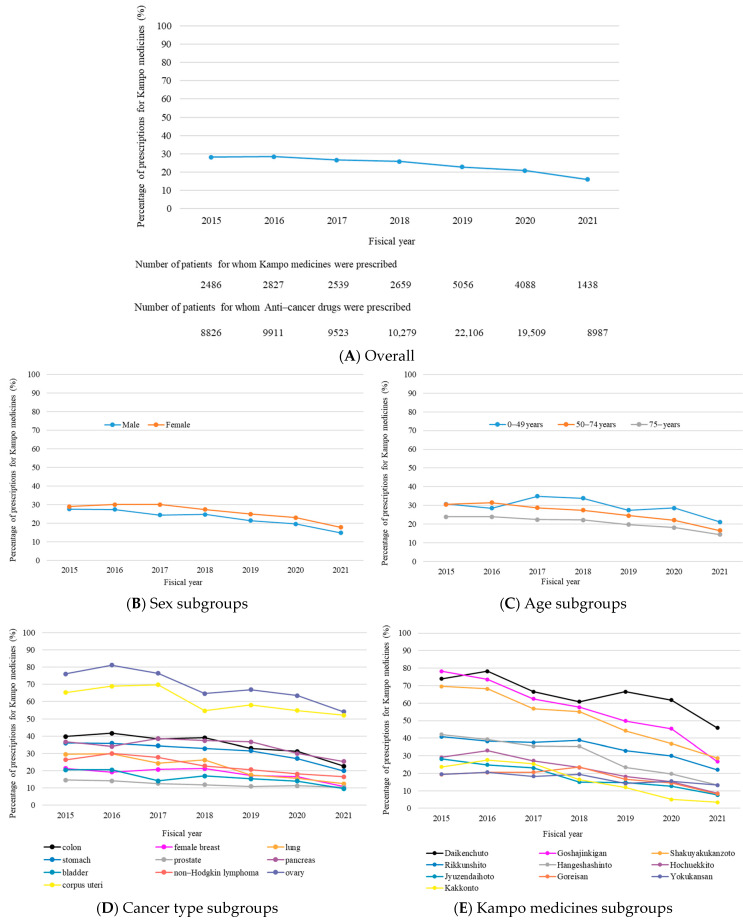
(**A**) Trends in the percentage of prescriptions of Kampo medicines among patients in the antineoplastic and immunomodulatory agent prescription periods; (**B**–**E**) Trends estimated using selected subgroup variables (sex (**B**), age (**C**), cancer type (**D**), and the top 10 Kampo medicines (**E**)) and the percentage of prescriptions of Kampo medicines among patients in the antineoplastic and immunomodulatory agent prescription periods.

The proportion of patients who were prescribed Kampo medicines during the anticancer treatment period decreased over time (*p* < 0.001). Among patients prescribed anticancer drugs in fiscal year 2015, approximately 28.2% were subsequently prescribed Kampo medicines, compared with approximately 16.0% in fiscal year 2021 (*p* value for trend < 0.001). This trend was consistent across subgroup analyses based on sex, age, cancer type, and the ten most frequently prescribed Kampo medicines during the anticancer treatment period (all *p* values for trend < 0.001) (Figure 2).

### 3.4. Top 10 Kampo Medicines

**Figure 3:**
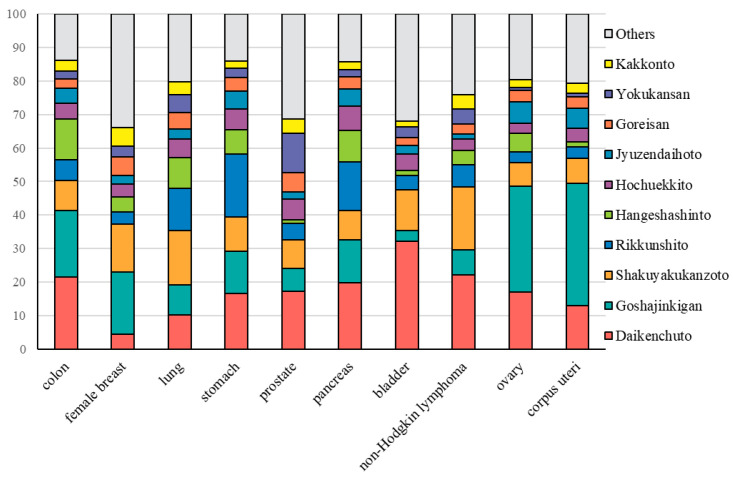
Percentage of prescriptions for Kampo medicine according to cancer type.

Figure 3 illustrates the most frequently prescribed Kampo medicines for the top 10 cancer types in the Kampo prescription group. Daikenchuto was the most commonly prescribed medicine for patients with colon, pancreatic, bladder, and prostate cancers, as well as non-Hodgkin lymphoma. Similarly, Goshajinkigan was the most frequently prescribed Kampo medicine for female patients with breast, ovarian, and corpus uteri cancers. For patients with lung cancer, Shakuyakukanzoto emerged as the most commonly prescribed drug, while Rikkunshito was prominently used for those with stomach cancer.

The top five anticancer drugs prescribed before Kampo medicines for each of the top 10 Kampo medicines are shown in Table S1. Among the patients prescribed Daikenchuto, the top five anticancer drugs were gemcitabine (895 patients, 19.0%), oxaliplatin (864 patients, 18.4%), cisplatin (787 patients, 16.7%), tegafur/gimeracil/oteracil (719 patients, 15.3%), and carboplatin (671 patients, 14.3%). For patients prescribed Goshajinkigan, the top five anticancer drugs were paclitaxel (1378 patients, 35.6%), carboplatin (1186 patients, 30.6%), oxaliplatin (1157 patients, 29.9%), capecitabine (628 patients, 16.2%), and fluorouracil (556 patients, 14.4%). Among patients prescribed Shakuyakukanzoto, the top five anticancer drugs were cisplatin (635 patients, 18.4%), carboplatin (572 patients, 16.5%), cyclophosphamide (539 patients, 15.6%), oxaliplatin (456 patients, 13.1%), and fluorouracil (450 patients, 13.0%). Among patients prescribed Rikkunshito, the top five anticancer drugs prescribed were tegafur/gimeracil/oteracil (590 patients, 25.0%), oxaliplatin (468 patients, 19.8%), cisplatin (451 patients, 19.1%), gemcitabine (363 patients, 15.3%), and fluorouracil (326 patients, 13.8%). Among patients prescribed Hangeshashinto, irinotecan (640 patients, 33.2%), oxaliplatin (562 patients, 29.1%), fluorouracil (532 patients, 27.6%), tegafur/gimeracil/oteracil (418 patients, 21.7%), and levofolinate calcium (412 patients, 21.4%) were the most commonly prescribed anticancer drugs.

For Daikenchuto, the most common diagnoses made between the initial prescription of anticancer drugs and Kampo medicine prescriptions included constipation, followed by abdominal bloating. Goshajinkigan was the most frequently prescribed medicine for patients with lower back pain and peripheral neuropathy. Shakuyakukanzoto and Rikkunshito were often used for constipation and chemotherapy-induced nausea and vomiting, whereas Hangeshashinto was prescribed for diarrhea and oral mucositis. The results for other Kampo medicines are presented in Table S2.

## 4. Discussion

To our knowledge, this is the first report on the utilization of Kampo medicine in patients with cancer receiving anticancer drugs using a nationwide claims database in Japan. Approximately 23.7% of patients were prescribed Kampo medicines during the anticancer treatment period. Previous studies examining Kampo medicine utilization among patients with cardiovascular disease and pregnant women reported usage rates of 8.9% and 48%, respectively [27,28]. The higher usage rate among pregnant women than among patients with cancer is believed to be due to the limited availability of Western medicine. The distribution of cancer types among patients prescribed anticancer drugs in this study closely matched the distribution of cancer incidence in Japan from 2016 to 2018, as reported by the Cancer Information Service of the National Cancer Center in Japan [29]. The proportion of Kampo medicine prescriptions varied according to cancer type. Patients with ovarian and corpus uteri cancers had high prescription rates of approximately 68.3% and 58.7%, respectively, whereas those with prostate and bladder cancers had low prescription rates of approximately 12.0% and 14.9%, respectively. This difference may be explained by existing studies evaluating the efficacy and safety of Kampo medicines for gynecological cancers, such as ovarian and corpus uteri cancers [30,31]. In contrast, few studies have evaluated the efficacy and safety of Kampo medicine in patients with prostate and bladder cancer. Furthermore, although no studies have evaluated the efficacy and safety of Kampo medicines during anticancer treatment for patients with non-Hodgkin lymphoma, the prescription rate was approximately 22.2%, indicating that patients with some cancer types utilized Kampo medicines without strong scientific evidence. Additionally, younger patients were found to be more likely to be prescribed Kampo medicines during cancer treatment. This may reflect differences in patient or physician preferences, as younger patients may seek complementary therapies to alleviate symptoms and maintain QOL during treatment.

The proportion of newly prescribed Kampo medicines during the anticancer treatment period decreased from fiscal year 2015 to fiscal year 2021. This decline was evident even after adjusting for clinical factors, such as sex, age, and cancer type. Additionally, an analysis of the trends for the top 10 most commonly prescribed Kampo medicines showed a consistent decline. A similar trend was observed in the use of Goshajinkigan for oxaliplatin-induced peripheral neuropathy from 2014 to 2020 [32]. This decline may reflect the influence of recent clinical trials on Kampo medicine efficacy among patients with cancer, which have yielded negative results. A systematic review of Goshajinkigan for chemotherapy-induced peripheral neuropathy (CIPN) found no evidence of efficacy [33]. Similarly, studies comparing Hangeshashinto with a placebo for chemotherapy-induced oral mucositis did not demonstrated its efficacy [34,35]. In contrast, Kampo medicine use among patients with cardiovascular disease during hospitalization increased over 12 years from 2010 to 2021, differing from the trend observed in this study [27]. No randomized controlled trials (RCTs) have evaluated the efficacy of Kampo medicine for the treatment of cardiovascular diseases. Kampo medicines used in patients with cardiovascular disease are likely to be used for the symptoms of other comorbid conditions present in these patients. In contrast, several RCTs have evaluated the efficacy of Kampo medicine in patients with cancer undergoing chemotherapy [30,36,37,38,39,40,41]. Therefore, physicians can make more evidence-based choices when selecting Kampo medicine for oncology symptoms compared to cardiovascular conditions. Trends in the usage rates of Kampo medicine are thought to be influenced by the presence or absence of scientific evidence regarding the efficacy of Kampo medicines. Additionally, older patients often have multiple comorbidities [42]; however, studies on patients with cardiovascular disease do not exclude those already using Kampo medicines before the observations began [27]. Therefore, the continuous use of Kampo medicine before the study period may have affected the results. However, our study excluded patients who used Kampo medicines for symptom relief before the initiation of chemotherapy, thereby limiting the inclusion of those using Kampo medicines for symptoms unrelated to anticancer treatments. This exclusion may have affected the results.

Among patients with cancer undergoing anticancer treatment, Daikenchuto was the most frequently prescribed Kampo medicine. RCTs have shown that Daikenchuto is effective in improving gastrointestinal motility in patients with colon and gastric cancer after surgery [36,37]. In this study, a comparison of patient backgrounds between those with and without Kampo prescriptions revealed that patients with colon and stomach cancers were significantly more likely to be prescribed Kampo medicines. However, the recommended duration of Daikenchuto use is approximately one week postoperatively [38]. In this study, we also examined patients who were prescribed Daikenchuto within seven days of the date of surgery. Among the 4692 patients who were prescribed Daikenchuto, 1098 (approximately 23.4%) received the prescription within seven days of the date of surgery. Conversely, no studies have assessed whether Daikenchuto is effective in alleviating the side effects of anticancer drugs. Therefore, many patients who were prescribed Daikenchuto in this study may have used it for symptoms unrelated to the anticancer treatment. Diagnoses made between the initial anticancer drug and Daikenchuto prescriptions most commonly included constipation, with magnesium oxide being frequently coprescribed, suggesting that Daikenchuto was used to treat constipation.

Goshajinkigan has demonstrated efficacy in RCTs for CIPN associated with oxaliplatin, paclitaxel/carboplatin, and docetaxel [30,39,40,41]. In our study, the top three anticancer drugs prescribed to patients using Goshajinkigan were paclitaxel, carboplatin, and oxaliplatin. Goshajinkigan is primarily prescribed for female patients with breast, ovarian, and corpus uteri cancers, with a predominant focus on gynecological cancers. The higher prevalence of patients with ovarian and corpus uteri cancers in the Kampo medicines prescription group may reflect this trend. Given that standard chemotherapy treatments for these cancers include paclitaxel, carboplatin, and oxaliplatin, it is conceivable that Goshajinkigan is frequently used to manage CIPN. However, the prescription rate decreased significantly during the study period. In 2017, the Clinical Guidelines for the Management of Chemotherapy-Induced Peripheral Neuropathy in Japan 2017 recommended only duloxetine for CIPN treatment, excluding Goshajinkigan [43]. A 2017 survey examining clinicians’ treatment choices for CIPN before and after the implementation of the Clinical Guidelines for the Management of Chemotherapy-Induced Peripheral Neuropathy in Japan found that duloxetine use increased between 2015 and 2019. In contrast, a significant decrease was observed in the use of Goshajinkigan, vitamin B12, and opioids, which are not recommended by the guidelines [43]. In this study, the publication of the guidelines may have influenced the decreasing trend in the use of Goshajinkigan among patients undergoing anticancer drug therapy. Yokoyama et al. reported that while the prescription rate of Goshajinkigan as a treatment for CIPN decreased, the prescription rates of Western medications such as duloxetine, pregabalin, and mirogabalin increased [42]. Furthermore, recent studies have suggested the potential efficacy of mirogabalin for CIPN symptom relief [44], contributing to the decreased use of Goshajinkigan. Diagnoses between the initial anticancer drug and Goshajinkigan prescriptions often included lower back pain (19.2%), suggesting some use for indications other than CIPN.

Shakuyakukanzoto is most commonly prescribed for patients with breast, lung, and colon cancers. RCTs have demonstrated their efficacy in relieving taxane-induced myalgia in patients with lung cancer undergoing carboplatin/paclitaxel therapy [45]. Additionally, studies have evaluated the efficacy of Shakuyakukanzoto for chemotherapy-induced hiccups in patients with lung cancer [46]. However, these studies were small and had limited evidence. To our knowledge, no study has evaluated its efficacy in patients with breast or colon cancers. Shakuyakukanzoto has the highest rate of reported side effects among Kampo medicines, particularly pseudoaldosteronism [47]. Risk factors include age (≥70 years), low body weight (<50 kg), and glycyrrhizin use for >14 days [48]. In this study, the median age of the patients who were prescribed Shakuyakukanzoto was 69 years, and the median prescription period was 17 days. In clinical trials using Shakuyakukanzoto to relieve taxane-induced muscle pain, the duration of use was 21 days, indicating a treatment period of ≥17 days [45]. Moreover, patients with cancer are prone to low body weight, and caution is warranted regarding the potential development of pseudoaldosteronism when using Shakuyakukanzoto in these patients.

Rikkunshito, Hochuekkito, and Jyuzendaihoto are used to treat patients with cancer cachexia [49]. Cancer cachexia is a complex metabolic disorder that leads to weight loss, appetite loss, fatigue, and decline in the QOL of patients with cancer. It is primarily observed in patients with pancreatic, lung, gastric, and colon cancer [50]. In this study, the cancers for which Rikkunshito, Hochuekkito, and Jyuzendaihoto were frequently prescribed included pancreatic, lung, gastric, and colon cancer. Although clinical trials have been conducted for Jyuzendaihoto for the treatment of cancer cachexia, its efficacy remains uncertain owing to differences in the definition of cachexia compared to the current general standards [51]. No trial has assessed the use of Rikkunshito or Hochuekkito for cachexia. In Japan, anamorelin, a ghrelin receptor agonist, was approved for the treatment of cancer cachexia in April 2021 [52,53]. Before the approval of anamorelin, no specific treatment was available for cachexia. Therefore, these Kampo medicines may have been used as CAM.

Hangeshashinto is frequently prescribed to patients undergoing chemotherapy regimens, including irinotecan, oxaliplatin, fluorouracil, tegafur-gimeracil-oteracil potassium, and levofolinate. The most commonly recorded conditions between the initiation of chemotherapy and the prescription of Hangeshashinto were diarrhea and oral mucositis. Clinical studies have evaluated the efficacy of Hangeshashinto in irinotecan-induced diarrhea (IID) [54]. Additionally, clinical studies have evaluated the efficacy of S-1 and fluorouracil regimens for oral mucositis. Nevertheless, the prescription rate for Hangeshashinto declined. In a systematic review of Hangeshashinto for IID, significant efficacy was demonstrated for severe diarrhea (National Cancer Institute Common Terminology Criteria for Adverse Events grade ≥ 3) [55]. However, the quality of the studies was considered low because of the short observation period. Furthermore, a correlation between high-grade IID incidence and UGT1A1 polymorphisms has been identified [56]. Despite this, no studies have investigated the efficacy of Hangeshashinto for IID while accounting for UGT1A1 polymorphisms. In clinical practice, the use of Hangeshashinto to prevent IID was initiated before irinotecan administration [54]. In this study, patients who had been prescribed Kampo medicines before their initial anticancer drug prescription were excluded; thus, patients using Hangeshashinto for IID prevention at the time of the first irinotecan prescription were not included. Therefore, it is possible that many of the patients in this study used Hangeshashinto for purposes other than IID prevention. Among patients undergoing chemotherapy, a potential use of Hangeshashinto other than for IID prevention includes relief from oral mucositis. However, the efficacy of Hangeshashinto for oral mucositis has not yet been demonstrated [34,35]. These findings may explain the observed decrease in the Hangeshashinto usage rate.

For patients with cancer, CAM plays a significant role in improving QOL and alleviating symptoms. However, the practice of evidence-based medicine is essential, and scientific validation is necessary to ensure the safety and efficacy of the treatments offered to patients. Although traditional treatments such as Kampo medicine have a long history of use, a key challenge is the lack of sufficient evaluation based on modern medical standards [23]. It is crucial to scientifically validate the effectiveness and safety of Kampo medicines to build robust evidence for their effective use as CAM. A survey of government research grants for Kampo medicine in Japan from 1997 to 2017 showed a continuous increase in the number of approved Kampo research projects and funding since 2010, suggesting that additional scientific evidence regarding the efficacy and safety of Kampo medicine will be established in the future [57]. This is expected to enhance its credibility in clinical settings and expand the treatment options for patients with cancer.

This study had certain limitations. First, determining the symptoms for which Kampo medicine was used proves difficult. Therefore, the results of this study reflect the actual usage of Kampo medicines prescribed to patients with cancer during the period of cancer drug treatment rather than the specific use of Kampo as supportive therapy for anticancer drugs. Second, we could not evaluate the efficacy or safety of Kampo medicines because of the nature of the database studies. Kampo medicines are often prescribed continuously without clear indications of symptom improvement, which makes it difficult to evaluate their impact through database studies. Future clinical trials focusing on frequently prescribed Kampo medicines are necessary. Third, we were unable to obtain data on over-the-counter Kampo medicine. Therefore, the proportion of patients with cancer who used Kampo medicines during the anticancer drug treatment period may have been underestimated in this study. Fourth, patient adherence could not be assessed using this database. Fifth, although this study revealed trends in the prescription of Kampo medicines among patients undergoing anticancer treatment, the detailed reasons driving these trends remain unclear. Future research should focus on identifying the underlying factors influencing Kampo medicine prescription trends, such as patient preferences, physician decision-making processes, and institutional policies, to provide a more comprehensive understanding of these patterns.

## 5. Conclusions

This study is the first report to analyze the utilization of Kampo medicines prescribed to patients undergoing anticancer drug treatment using a nationwide database. Approximately 23.7% of patients were prescribed Kampo medicines during their anticancer treatment period, with a decreasing trend in prescriptions observed across Japan. Younger patients and those with gastrointestinal cancers, such as colon and gastric cancer, or gynecological cancers were more likely to use Kampo medicines during anticancer treatment. However, the decreasing trend in the prescription of Kampo medicines was consistently observed regardless of sex, age, or cancer type.

Traditionally, Kampo medicines have been widely used for symptom relief and may significantly aid in managing the side effects of cancer treatments. However, the lack of evidence based on modern medical standards remains a challenge. To enhance patients’ QOL and ensure safety, it is essential to scientifically validate the efficacy and safety of Kampo medicines, thereby advancing their role in cancer supportive care.

## Figures and Tables

**Table 1:** Patient background characteristics.

	Total, n (%)	Kampo Prescriptions During Anticancer Drug Treatment		Absolute Standardized Difference ***
		Yes, n (%)	No, n (%)	
	89,141	21,093	68,048	
Male sex	52,532 (58.9)	11,689 (55.4)	40,843 (60.0)	0.093
Age: median [IQR *]	72 [64–79]	70 [62–77]	72 [65–79]	
Age group				0.164
0–49 years	6322 (7.1)	1842 (8.7)	4480 (6.6)	
50–74 years	47,196 (52.9)	12,044 (57.1)	35,152 (51.7)	
≥75 years	35,623 (40.0)	7207 (34.2)	28,416 (41.8)	
Type of carcinomas				
prostate	13,070 (14.7)	1567 (7.4)	11,503 (16.9)	0.293
female breast	13,030 (14.6)	2370 (11.2)	10,660 (15.7)	0.13
colon	10,693 (12.0)	3717 (17.6)	6976 (10.3)	0.214
lung	10,655 (12.0)	2211 (10.5)	8444 (12.4)	0.061
bladder	9484 (10.6)	1417 (6.7)	8067 (11.9)	0.178
stomach	6109 (6.9)	1913 (9.1)	4196 (6.2)	0.11
non-Hodgkin lymphoma	5378 (6.0)	1193 (5.7)	4185 (6.2)	0.021
pancreas	4248 (4.8)	1445 (6.9)	2803 (4.1)	0.12
liver	3323 (3.7)	657 (3.1)	2666 (3.9)	0.044
leukemia	2064 (2.3)	441 (2.1)	1623 (2.4)	0.02
multiple myeloma	1407 (1.6)	413 (2.0)	994 (1.5)	0.038
esophagus	1396 (1.6)	356 (1.7)	1040 (1.5)	0.013
corpus uteri	1338 (1.5)	785 (3.7)	553 (0.8)	0.196
ovary	1322 (1.5)	903 (4.3)	419 (0.6)	0.239
gallbladder	1306 (1.5)	290 (1.4)	1016 (1.5)	0.01
kidney	1005 (1.1)	319 (1.5)	686 (1.0)	0.045
cervix uteri	917 (1.0)	472 (2.2)	445 (0.7)	0.133
brain, central nervous system	605 (0.7)	154 (0.7)	451 (0.7)	0.008
larynx	222 (0.3)	80 (0.4)	142 (0.2)	0.032
lip, oral cavity	214 (0.2)	56 (0.3)	158 (0.2)	0.007
hypopharynx	211 (0.2)	77 (0.4)	134 (0.2)	0.032
NMSC **	184 (0.2)	18 (0.1)	166 (0.2)	0.039
oropharynx	172 (0.2)	66 (0.3)	106 (0.2)	0.033
thyroid	171 (0.2)	21 (0.1)	150 (0.2)	0.03
testis	148 (0.2)	34 (0.2)	114 (0.2)	0.002
mesothelioma	147 (0.2)	27 (0.1)	120 (0.2)	0.012
Hodgkin lymphoma	109 (0.1)	23 (0.1)	86 (0.1)	0.005
melanoma of skin	71 (0.1)	17 (0.1)	54 (0.1)	<0.001
salivary glands	50 (0.1)	10 (0.0)	40 (0.1)	0.005
nasopharynx	49 (0.1)	26 (0.1)	23 (0.0)	0.032
vulva	23 (0.0)	9 (0.0)	14 (0.0)	0.012
penis	13 (0.0)	3 (0.0)	10 (0.0)	<0.001
vagina	5 (0.0)	2 (0.0)	3 (0.0)	0.006
Kaposi sarcoma	2 (0.0)	1 (0.0)	1 (0.0)	0.006

* IQR, interquartile range ** NMSC, non-melanoma skin cancer. *** An absolute standardized difference >0.10 indicates a significant difference between the groups.

**Table 2:** Background of patients for whom Kampo medicine was prescribed.

	Total, n (%)
	21,093
Male sex	11,689 (55.4)
Age: median [IQR *]	70 [62–77]
Number of days from the date of anticancer drug prescription to the date of Kampo medicine prescription: median [IQR *]	75 [14–209]
Top 10 Kampo medicines	
Daikenchuto	4692 (22.2)
Goshajinkigan	3866 (18.3)
Shakuyakukanzoto	3458 (16.4)
Rikkunshito	2360 (11.2)
Hangeshashinto	1925 (9.1)
Hochuekkito	1429 (6.8)
Jyuzendaihoto	1157 (5.5)
Goreisan	1153 (5.5)
Yokukansan	1086 (5.1)
Kakkonto	965 (4.6)
Top 10 types of carcinomas	
colon	3717 (17.6)
female breast	2370 (11.2)
lung	2211 (10.5)
stomach	1913 (9.1)
prostate	1567 (7.4)
pancreas	1445 (6.6)
bladder	1417 (6.7)
non-Hodgkin lymphoma	1193 (5.7)
ovary	903 (4.3)
corpus uteri	785 (3.7)

* IQR, interquartile range.

## Data Availability

The data presented in this study are available on request from the corresponding author.

## Supplementary Materials

The following supporting information can be downloaded at: https://www.mdpi.com/article/10.3390/curroncol32020100/s1, Table S1: Top 5 most commonly prescribed anticancer drugs used before prescribing the top 10 Kampo medicines; Table S2: Top 10 Kampo medicines and frequent diagnoses.
