# Supplementary material for: Trends in Kampo Medicine Usage as Supportive Care During Anticancer Drug Treatment in Japanese Patients: A Nationwide Cohort Analysis from Fiscal Years 2015 to 2021
Source: Curr Oncol. 2025 Feb 10;32(2):100. doi: 10.3390/curroncol32020100 (PMC11854802; doi:10.3390/curroncol32020100)
Supplement: Supplementary file 1 [file curroncol-32-00100-s001.zip › curroncol-3424502-supplementary.pdf]

Table S1. Top 5 most commonly prescribed anticancer drugs used before prescribing the top 10 Kampo medicines

Top 10 Kampo medicines	n	Frequently the diagnoses, n (%)									
		First most frequently used type		Secound most frequently used type		Third most frequently used type		Fourth most frequently used type		Fifth most frequently used type	
Daikenchuto	4692	Gemcitabine	895 (19.0)	Oxaliplatin	864 (18.4)	Cisplatin	787 (16.7)	TS-1*	719 (15.3)	Carboplatin	671 (14.3)
Goshajinkigan	3866	Paclitaxel	1378 (35.6)	Carboplatin	1186 (30.6)	Oxaliplatin	1157 (29.9)	Capecitabine	628 (16.2)	Fluorouracil	556 (14.4)
Shakuyakukanzoto	3458	Cisplatin	635 (18.4)	Carboplatin	572 (16.5)	Cyclophos-phamide	539 (15.6)	Oxaliplatin	456 (13.1)	Fluorouracil	450 (13.0)
Rikkunshito	2360	TS-1*	590 (25.0)	Oxaliplatin	468 (19.8)	Cisplatin	451 (19.1)	Gemcitabine	363 (15.3)	Fluorouracil	326 (13.8)
Hangeshashinto	1925	Irinotecan	640 (33.2)	Oxaliplatin	562 (29.1)	Fluorouracil	532 (27.6)	TS-1*	418 (21.7)	Levofolinate calcium	412 (21.4)
Hochuekkito	1429	TS-1*	286 (20.0)	Oxaliplatin	232 (16.2)	Gemcitabine	216 (15.1)	Carboplatin	213 (14.9)	Cisplatin	196 (13.7)
Jyuzendaihoto	1157	TS-1*	242 (20.9)	Carboplatin	241 (20.8)	Oxaliplatin	233 (20.1)	Paclitaxel	214 (18.5)	Cisplatin	177 (15.3)
Goreisan	1153	Carboplatin	189 (16.4)	TS-1*	164 (14.2)	Paclitaxel	159 (13.8)	Docetaxel	151 (13.1)	Cyclophos-phamide	148 (12.8)
Yokukansan	1086	Bicalutamide	174 (16.0)	Cisplatin	111 (10.2)	TS-1*	110 (10.1)	Carboplatin	107 (9.9)	Oxaliplatin	94 (8.7)
Kakkonto	965	Carboplatin	144 (14.9)	Cyclophos-phamide	133 (13.8)	TS-1*	123 (12.7)	Paclitaxel	120 (12.4)	Oxaliplatin	111 (11.5)

*TS-1:Tegafur/Gimeracil/Oteracil

Table S2. Top 10 Kampo medicines and frequently the diagnoses

Top 10 Kampo medicines	n	Frequently the diagnoses, n (%)									
		First most frequently the diagnoses		Secound most frequently the diagnoses		Third most frequently the diagnoses		Fourth most frequently the diagnoses		Fifth most frequently the diagnoses	
Daikenchuto	4692	Constipation	1137 (24.2)	Flatulence and related conditions	880 (18.8)	Nausea and vomiting	732 (15.6)	Dehydration	509 (10.8)	Low back pain	499 (10.6)
Goshajinkigan	3866	Low back pain	742 (19.2)	Polyneuropathy, unspecified	665 (17.2)	Nausea and vomiting	630 (16.3)	Constipation	616 (15.9)	Xerosis cutis	357 (9.2)
Shakuyakukanzoto	3458	Constipation	549 (15.9)	Nausea and vomiting	481 (13.9)	Cramp and spasm	466 (13.5)	Low back pain	435 (12.6)	Insomnia	338 (9.8)
Rikkunshito	2360	Constipation	446 (18.9)	Nausea and vomiting	442 (18.7)	Anorexia	402 (17.0)	Dehydration	341 (14.4)	Chronic gastritis	308 (13.1)
Hangeshashinto	1925	Diarrhea	386 (20.1)	Stomatitis	381 (19.8)	Nausea and vomiting	371 (19.3)	Constipation	314 (16.3)	Dehydration	240 (12.5)
Hochuekkito	1429	Anorexia	485 (33.9)	Constipation	263 (18.4)	Nausea and vomiting	246 (17.2)	Low back pain	188 (13.2)	Dehydration	173 (12.1)
Jyuzendaihoto	1157	Anorexia	295 (25.5)	Constipation	173 (15.0)	Nausea and vomiting	169 (14.6)	Low back pain	127 (11.0)	Dehydration	112 (9.7)
Goreisan	1153	Constipation	223 (19.3)	Nausea and vomiting	190 (16.5)	Diarrhea	169 (14.7)	Low back pain	165 (14.3)	Dehydration	163 (14.1)
Yokukansan	1086	Insomnia	312 (28.7)	Constipation	197 (18.1)	Dehydration	138 (12.7)	Low back pain	120 (11.0)	Pain	119 (11.0)
Kakkonto	965	Cold	399 (41.3)	Acute upper respiratory infection	203 (21.0)	Constipation	184 (19.1)	Low back pain	146 (15.1)	Nausea and vomiting	134 (13.9)
